# The Impact of UK Medical Students’ Demographics and Socioeconomic Factors on Their Self-Reported Familiarity With the Postgraduate Training Pathways and Application Process: Cross-Sectional Study

**DOI:** 10.2196/49013

**Published:** 2023-11-24

**Authors:** Kaveh Davoudi, Tushar Rakhecha, Anna Chiara Corriero, Kar Chang Natalie Ko, Roseanne Ismail, Esther R B King, Linda Hollén

**Affiliations:** 1 Bristol Royal Infirmary University Hospital Bristol and Weston Bristol United Kingdom; 2 University of Bristol Medical School University of Bristol Bristol United Kingdom; 3 Anglia Ruskin Medical School Chelmsford United Kingdom; 4 National Health Service Gloucestershire Trust Gloucester United Kingdom; 5 University of Bristol Bristol United Kingdom

**Keywords:** age, career progression, career progression, clinicians, cross-sectional study, demographics, ethnicity, gender, leadership, medical students demographics, medical students, online survey, research, students, teaching, training

## Abstract

**Background:**

UK medical graduates can apply for specialty training after completing a 2-year internship (foundation training). Postfoundation training application requirements vary depending on specialty but fundamentally require key skills such as teaching, research, and leadership.

**Objective:**

This study investigated whether medical student demographics impact their self-reported familiarity with the Post-Foundation Training Pathways (PFTPs) and Post-Foundation Application Process (PFAP).

**Methods:**

This was a cross-sectional study using a Bristol Online Survey. We invited all UK medical students to answer a range of questions about their demographics. Students were then asked to rank their familiarity with PFTPs and PFAP on a scale of 1 to 5 (1=least familiar and 5=most familiar). The responses were collected between March 2022 and April 2022 and exported for further analysis. Statistical analysis was conducted in Stata (version 17.1; StataCorp) using chi-square tests.

**Results:**

A total of 850 students from 31 UK medical schools took part. There was a significant difference between gender and self-reported familiarity with PFTPs (*P*<.001) and PFAP (*P*<.001), with male students expressing higher familiarity. Similarly, there was a difference between ethnicity and self-reported familiarity with PFTPs (*P*=.02) and PFAP (*P*<.001), with White students more likely to express higher familiarity than their Black, Asian, or Mixed Ethnic counterparts. Lastly, there was an overall difference between medical background and age and self-reported familiarity with PFTPs and PFAP (all *P*<.001), with students from medical backgrounds and older students being more likely to express higher familiarity.

**Conclusions:**

The impact of gender, ethnicity, age, and medical background on students’ self-reported familiarity with PFTPs and PFAP is significant. Further studies are required to evaluate the impact of these factors on tested knowledge of PFTPs and PFAP and whether this impacts the success rate of postfoundation applications.

## Introduction

In the United Kingdom, medical training starts with students spending between 4 and 6 years at the undergraduate level. Following this, all UK medical students must complete the foundation training program, a 2-year paid internship rotating through 6 different placements, before starting specialty training [1].

Postfoundation training lasts between 3 and 8 years, with many doctors taking years out of training before entering a specialty training program. Recruitment to specialty training programs varies between specialties. It might include a combination of a Multi-Specialty Recruitment Assessment (MSRA) exam score [2], a portfolio [3], as well as interviews.

Candidates can often prepare for the MSRA and the interview components of these postgraduate recruitments in the months leading up to the start of the application cycle. However, the portfolio part of the selection process often takes many years to develop. The portfolio includes components such as teaching experience, involvement in research, taking on leadership roles, and additional qualifications. The longer preparation time for those components could create an unfair advantage for candidates who know about the application process earlier in their undergraduate careers and, in turn, limit candidates’ specialty selection.

The need for a diverse, well-balanced medical workforce is an established concept. Numerous studies have shown improved outcomes when patients match the gender and ethnicity of their physicians [4,5]. A diverse medical workforce is essential for patients and good for bringing a range of experiences together, paving the way for innovation and improvement of services. The primary aim of this study was to investigate any difference between medical students’ demographics and their self-reported familiarity with Post-Foundation Training Pathways (PFTPs) and Post-Foundation Application Process (PFAP). The secondary aim was to investigate the difference between demographics and training pathway choices.

## Methods

### Study Design and Compiling the Questionnaire

This was a cross-sectional study using a web-based questionnaire. Bristol Online Surveys (University of Bristol) was used to collect responses.

The authors designed the survey to include questions about the participants’ demographics, including gender, ethnicity, medical background, age, and training stage, as well as questions on self-reported familiarity with PFTPs and PFAPs. The questionnaire also assessed participants’ preferred training programs out of a selection of common pathways, including acute care common stem (ACCS), core surgical training (CST), general practice, internal medical training (IMT), neurosurgery, obstetrics and gynecology, ophthalmology, psychiatry, and radiology. The survey involved a range of question styles, including Likert scale, multiple choice, and free text (Multimedia Appendix 1).

This was an open, voluntary survey, and participants were required to complete all the questions before being able to submit their responses. When relevant, participants were given response options such as “not applicable” or “rather not say.” The Bristol Online Surveys prevented participants from submitting multiple responses using web-based cookies.

### Recruitment Process

All current UK medical students were eligible to take part. To maximize the reach of the questionnaire, local collaborators were recruited from several universities across the country to promote the study and assist in collecting responses (see “Acknowledgments” section). The collaborators and authors carried out a trial run to ensure the functionality and usability of the electronic questionnaire before the national opening.

This study was advertised through social media channels as well as locally placed printed posters. In order to incentivize participation, students were offered a chance to enter a draw to win a £50 (US $61) Amazon voucher, which was self-funded by the authors. If the participants opted for entry into the draw, they were asked to enter their email address for the prize allocation. This was collected separately and not linked to the rest of the questionnaire. After prize allocation, all email addresses were deleted.

The data collection took place over 2 months, between March 2022 and April 2022. The data were stored safely on the Bristol Online Survey’s servers and was anonymously exported for further analysis.

### Definitions

The following definitions were given to the participants to standardize their answers.

Coming from a medical background was defined as having a family member or close friend with a medical degree.

Self-reported familiarity with PFTPs was defined as understanding the number of years involved in the desired training pathway and whether the training pathway was run through or required multiple applications (eg, 2 years of CST followed by another application cycle for 4-5 years of higher surgical training).

Self-reported familiarity with PFAP was defined as an understanding of the current criteria for candidate selection (eg, use of MSRA, portfolio, and interviews) and the content of the said criteria (eg, portfolio and interviews assessing qualities such as leadership, academics, and teaching).

### Analysis

For familiarity with PFTPs and PFAP, respondents were asked to express their responses based on a 1-5–point Likert scale, which ranged from the lowest level of familiarity (1) to the highest level of familiarity (5), with the average response being in the middle. Respondents were also asked to indicate what their preferred training pathway after the foundation program would be if they were to choose at this point in time.

The data were analyzed using Stata (version 17.1; StataCorp). Overall differences between demographics and self-reported familiarity were analyzed using chi-square tests. As our contingency tables are larger than 2 by 2, we then used Pearson-adjusted residuals to examine where any significant differences lie. A residual is the difference between the observed and expected values for a cell. The larger the residual, the greater the contribution to the overall chi-square value and significance. Adjusted residuals are Pearson residuals divided by an estimate of their SE, *P*<.05 represents residuals of ≥1.96 (indicated by relevant footnotes in Tables 1-3), and a more conservative *P*<.01 represents residuals of ≥2.58 (indicated by relevant footnotes in Tables 1-3).

**Table 1:** Illustrates the Pearson residual values for students self-reported familiarity with Post-Foundation Training Pathways and asks the question “How familiar are you with training pathways of doctors after foundation training?” Chi-square and associated *P* values refer to overall significance between the variables tested and the Pearson adjusted residuals illustrate where significant differences lie.

Variables			Self-reported familiarity with Post-Foundation Training Pathways out of 5 (1=least familiar and 5=most familiar)											Chi-square (*df*)			*P* value
			1		2		3		4		5						
**Sex**														54.6 (4)			<.001
	Female	2.13^a^		4.41^b^		–2.07^a^		–4.22^b^		–4.50^b^							
	Male	–2.13^a^		–4.41^b^		2.07^a^		4.22^b^		4.50^a^							
**Race or ethnicity**														20.1 (4)			<.001
	White	–1.65		–1.62		–0.16		3.75^b^		2.01^a^							
	BAME^c^	1.65		1.62		0.16		–3.75^b^		–2.01^a^							
**Medical background**														76.2 (4)			<.001
	No medical background	4.53^b^		4.01^b^		–3.12^b^		–6.31^b^		–2.88^b^							
	Medical background	–4.53^b^		–4.01^b^		3.12^b^		6.31^b^		2.88^b^							
**Mean age**														81.2 (4)			<.001
	<mean age	5.09^b^		3.85^b^		–4.51^b^		–3.96^b^		–4.88^b^							
	≥mean age	–5.09^b^		–3.85^b^		4.51^b^		3.96^b^		4.88^b^							

^a^*P*<.05.

^b^*P*<.001.

^c^BAME: Black, Asian, or Mixed Ethnic.

**Table 2:** Illustrates the self-reported familiarity with Post-Foundation Application Process, and asks the question “How familiar are you with training pathways of doctors after foundation training?” Chi-square and associated *P* values refer to overall significance between the variables tested and the Pearson adjusted residuals illustrate where significant differences lie.

Variables			Self-reported familiarity with Post-Foundation Application Process out of 5 (1=least familiar and 5=most familiar)											Chi-square (*df*)			*P* value
			1		2		3		4		5						
**Sex**														27.4 (4)			<.001
	Female	–1.03		4.56^a^		0.18		–3.03^a^		–2.07^b^							
	Male	1.03		–4.56^a^		–0.18		3.03^a^		2.07^b^							
**Race or ethnicity**														11.4 (4)			.02
	White	–1.13		–2.85^a^		2.25^b^		0.83		0.63							
	BAME^c^	1.13		2.85^a^		–2.25^b^		–0.83		–0.63							
**Medical background**														52.6 (4)			<.001
	No medical background	2.96^a^		4.57^a^		0.19		–6.05^a^		–1.40							
	Medical background	–2.96^a^		–4.57^a^		–0.19		6.05^a^		1.40							
**Mean age**														47.0 (4)			<.001
	<mean age	3.58^a^		3.81^a^		–0.17		–4.33^a^		–3.34^a^							
	≥mean age	–3.58^a^		–3.81^a^		0.17		4.33^a^		3.34^a^							

^a^*P*<.001.

^b^*P*<.05.

^c^BAME: Black, Asian, or Mixed Ethnic.

**Table 3:** Illustrates the training pathway choices and asks the question, “If you were to choose at this point in time, what is your most preferred training pathway to undertake after foundation training?” Chi-square and associated *P* values refer to overall significance between the variables tested and the Pearson adjusted residuals illustrate where significant differences lie.

Variables		Students’ choices of some of the common carrier pathways available after foundation training										Chi-square (*df*)			*P* value	
		Acute care common stem	Core surgical training	General practice training	Internal medical training	Neurosurgery	Obstetrics and gynecology	Ophthalmology	Psychiatry	Radiology						
**Sex**													35.5 (8)			<.001
	Female	0.60	–0.50	–0.43	0.51	–2.10^a^	3.86^b^	–0.88	0.49	–4.01^b^						
	Male	–0.60	0.50	0.43	–0.51	2.10^a^	–3.86^b^	0.88	–0.49	4.01^b^						
**Race or ethnicity**													27.6 (8)			.001
	White	4.06^b^	–1.05	–1.53	0.41	0.72	0.38	–1.15	–1.29	–2.72^b^						
	BAME^c^	–4.06^b^	1.05	1.53	–0.41	–0.72	–0.38	1.15	1.29	2.72^b^						
**Medical background**													15.7 (8)			.05
	No medical background	0.17	–1.49	0.65	–0.81	–1.67	2.96^b^	1.25	–0.50	0.75						
	Medical background	–0.17	1.49	–0.65	0.81	1.67	–2.96^b^	–1.25	0.50	–0.75						
**Mean age**													4.95 (8)			.08
	<mean age	–1.19	1.59	–0.65	–0.16	0.50	0.13	–0.32	–0.52	0.86						
	≥mean age	1.19	–1.59	0.65	0.16	–0.50	–0.13	0.32	0.52	–0.86						

^a^*P*<.05.

^b^*P*<.001.

^c^BAME: Black, Asian, or Mixed Ethnic.

### Ethical Considerations

The project was reviewed by the Faculty of Health Science Research Ethics Committee at the University of Bristol, and ethical approval was also granted (reference number: 9858). All participants were informed about the aims of the study, the average length of time required to fill out the survey, and the members of the investigating team. This information was included in all promotional material about the survey as well as the first page of the web-based survey. All the data were collected anonymously.

## Results

### Overview

A total of 850 UK medical students from over 31 medical schools completed the web-based questionnaire. IMT, CST, general practice training, and ACCS were the most popular training pathways that students expressed an interest in at this point of their university training (24.8%, 19.6%, 18.6%, and 14.8%, respectively).

### Gender

A total of 584 female, 245 male, and 11 nonbinary, nonconforming students took part. Also, 10 participants preferred not to declare their gender. Due to small sample sizes, the nonbinary, nonconforming students, and those who preferred not to mention their gender were excluded from further gender-based analyses. There was an overall significant difference between gender and self-reported familiarity with PFTPs (*P*<.001), where 40% (98/245) of the male students expressed high familiarity (4 or 5 on the Likert scale), compared to 26.5% (155/584) for female students (Figure S1-A in Multimedia Appendix 2 and Table 1). Similarly, there was an overall significant difference between male and female medical students and their self-reported familiarity with the PFAP (*P*<.001), where 67.1% (392/584) of the female students expressed low familiarity (1 or 2) compared to 44.1% (108/245) for male students. Significantly more male students expressed a higher familiarity (4 or 5) with the application process (male: 26.5% vs female: 10.3%; Figure S1-B in Multimedia Appendix 2; Table 2).

Lastly, there was a significant overall difference between genders and chosen training pathway (*P<*.001), where male students were more likely to choose radiology and neurosurgery training and female students more likely to choose obstetrics and gynecology as their potential training pathway (Figure S1-C in Multimedia Appendix 2 and Table 3).

### Ethnicity

A total of 458 students were of White ethnicity, while 377 were of Black, Asian, or Mixed Ethnic (BAME) backgrounds. Furthermore, 15 participants selected unknown or preferred not to declare their ethnicity. Due to a small sample size, this group was excluded from further ethnicity-based analyses. There was a small overall significant difference between self-reported familiarity with PFTPs and ethnicity (*P*=.02; Figure S2-A in Multimedia Appendix 2). BAME students were more likely to choose a 2 and less likely to choose a 3 on the Likert scale compared to White students (Table 1). There was a stronger and more significant difference between White and BAME students and their self-reported familiarity with PFAP. White students reported to be more familiar with PFAP (4 or 5) compared to their BAME counterparts (White: 19.8% vs BAME: 9%; Figure S2-B in Multimedia Appendix 2; Table 2).

When asked about training pathways that the students might choose at the time of the survey, there was a significant overall difference between White and BAME students (*P<*.001), where more White students (19.7% vs 9.5%) chose ACCS. This was reversed for radiology (White: 1.1% vs BAME: 4%), although the overall numbers for radiology were low (20 out of 850). There was no significant difference between the other training pathways and ethnicity (Figure S2-C in Multimedia Appendix 2 and Table 3).

### Medical Background

A total of 255 out of 850 students came from a medical background, defined as having a family member or close friend with a medical degree. There was a significant difference between medical background and self-reported familiarity with PFTPs (*P*<.001). A significantly higher proportion of students with no medical background expressed low familiarity (1 or 2) with the training pathway (no medical background: 41.1% vs medical background: 20%). Those with a medical background were also much more likely to answer a 4 (Figure 3A-A in Multimedia Appendix 2 and Table 1).

There was also a significant difference between coming from a medical background and self-reported familiarity with PFAP (*P*<.001). A higher proportion of students with no medical background expressed low familiarity (1 or 2) with the application process (no medical background: 69.1% vs medical background: 40%). Comparably, more students from a medical background indicated higher familiarity (4 or 5) with the application process (no medical background: 9.2% vs medical background: 28.2%; Figure 3-B in Multimedia Appendix 2; Table 2).

A total of 85% (217/255) of those who had come from a medical background thought that this had a positive impact on their familiarity with PFTPs and PFAPs.

There was no overall significant difference with medical background and selection of the training pathways (*P*=.05), except for obstetrics and gynecology (no medical background: 9.4% vs medical background 3.5%; Figure 3-C in Multimedia Appendix 2; Table 3).

### Age

The participants were from various training stages (71 in first year, 191 in second year, 245 in third year, 100 in fourth year, 113 in fifth year, 38 in sixth year, and 34 intercalating). The mean age of the participants was 22.5 years. The participants were categorized into 2 cohorts for the purposes of analysis: younger than the mean age (503 students) and at or older than the mean age (346 students). A significant difference (*P*<.001) can be seen between participants younger and older than their average age and their self-reported familiarity with PFTPs. A greater proportion of participants younger than the average age expressed lower familiarity (1 or 2) with the training pathway compared to those aged above average (<22.5 years=42.5% vs ≥ 22.5 years=23.7 %), with older respondents reporting higher familiarity (4 or 5) with the training pathway (<22.5 years=23.1% vs ≥22.5 years=41.3%; Figure 4-A in Multimedia Appendix 2; Table 1).

Results also showed a significant difference between age and PFAP (*P<.*001), where participants younger than the average age reported being less familiar (1 or 2) with PFAP (<22.5 years=72% vs ≥22.5 years=43.7%). In parallel, fewer participants with a younger age than average expressed higher familiarity (4 or 5) than those who aged above average (<22.5 years=8.9% vs ≥22.5 years=23.7%; Figure 4-B in Multimedia Appendix 2; Table 2). There was no significant difference (*P*=.76) between training pathway selection and age groups (Figure 4-C in Multimedia Appendix 2 and Table 3).

### Future Resources

Participants were also asked about their preferred method of further guidance on the application processes, where 72.9% (612/850) chose a website explaining all the training pathways and the application processes, 44.5% (378/850) selected mentorship schemes, 45.1% (383/850) chose short videos, and a third (281/850, 33.1%) chose lectures.

## Discussion

### Overview

Female students reported lower familiarity with the application process than male students. Numerous studies have demonstrated the lower number of women in academic medicine [6], surgical specialties [7], and leadership roles [8] in health care. This is despite a higher proportion (54% in 2019) of female doctors registering with the General Medical Council each year [9].

However, this high proportion of female medical students currently does not translate into a high proportion of female consultants, who only make up 36% of the consultant population [10]. Although this number has improved (30% in 2009), the rate of improvement has been very slow. Moreover, this number is much lower in historically male-dominated specialties such as surgery (14.7% in 2022) [11].

The lower self-reported familiarity with the training pathways and the application process could contribute to this lower rate of progression, especially in training pathways such as CST, where there is a strong emphasis on portfolio building in the years leading to the application. One of the factors contributing to this is the lack of senior female role models and mentors who would be able to advise the students throughout their medical school [12]. Implicit bias by seniors could also lead to an uneven distribution of portfolio-building opportunities [13].

The BAME medical students reported being less familiar with the PFAP than students from a White ethnic background. The proportion of UK graduates from a BAME background registering with the General Medical Council was 23% in 2019 [9]. A great amount of work has been done to increase the number of successful applications to medical schools for students from non-White ethnic backgrounds. The Medical School Council reported an increase of 58% in students of Black heritage in 2019 [14].

Many widening participation schemes have been designed to provide opportunities such as voluntary work, shadowing placement, and interview practice, among others, for pupils from diverse ethnic and socioeconomic backgrounds. However, most support programs stop after entry into medical school. This could be a contributing factor to the lower self-reported familiarity of students from BAME. Similar to gender, the lower representation of BAME doctors in senior roles translates into a lower number of role models, which could be affecting the students’ overall familiarity with PFAP.

Almost a third (255/850, 30%) of the students reported being from a medical background, and the majority of those (217/255, 85%) believed that this had a positive impact on their self-reported familiarity with PFTPs and PFAP. This was reflected in the familiarity responses, where students from medical backgrounds reported higher familiarity with PFAP. These results show that being from a medical background could have an impact even after entry into medical school. Medical students would be able to learn about the requirements of PFAP early on through their medical contacts, enabling them to get involved in extracurricular activities such as teaching, research, and presentations earlier than their peers. The subject of the heritability of medicine is a concept that has been introduced previously. A study on 3 generations of Swedish physicians found that the proportion of physicians with one parent from a medical background rose from 6% in the 1950s to 20% in 1980 [15]. They hypothesized factors such as financial background as important contributors to this rise in medical students from medical backgrounds. However, this study suggests that the role of nonfinancial advantages cannot be ignored, as it was demonstrated that a medical background is correlated with significantly higher self-reported familiarity with PFAP.

Lastly, we analyzed the impact of age on the students’ self-reported familiarity. The different lengths of medical degree courses (4-year, 5-year, and 6-year courses) as well as the possibility of intercalating at different stages throughout the medical school do not allow direct comparison between training years. As expected, the self-reported familiarity with PFTP and PFAP is different between age groups. As the students spend more time in the clinical setting by completing their clinical years, they are more likely to become familiar with PFTPs and PFAP. Furthermore, they are also more likely to come across mentors who can guide them through the process.

There are clear differences between gender, ethnic background, and medical background, and medical students’ self-reported familiarity with PFTPs and PFAP. It is unclear how early self-reported familiarity with the PFTP and PFAP impacts career progression and success in postgraduate training. However, assuming early self-reported familiarity correlates to actual familiarity and, in turn, an advantage, this may be a predisposing factor to some of the discrepancies observed in the demographic composition of the general population and the senior doctor community.

Traditionally, medical schools are thought to be places that prepare students for the early years after graduation. However, certain aspects of postgraduate training applications, such as leadership, teaching, and research, take time to develop. The earlier the students know about these requirements, the more likely they are to be able to seek opportunities to develop those skills in time for postgraduate applications. Addressing this gap in the undergraduate curriculum requires national collaboration from medical schools and Royal Colleges to develop resources and signpost students to them. As suggested by the students, this could take the form of one website with all the required information, a series of short videos explaining the PFAP, as well as mentoring schemes.

This study had several limitations. First, low numbers in smaller specialties such as radiology and neurosurgery make it difficult to draw any conclusions about these specialties. Similarly, low numbers of nonbinary, nonconforming students mean no statistically significant conclusions can be drawn. Lastly, we used candidates self-reported familiarity. Future studies should consider using objective measures of familiarity by evaluating knowledge through questionnaires.

### Conclusions

Self-reported familiarity with PFTPs and PFAP differs significantly based on gender, ethnicity, medical background, and age. It is unclear whether early familiarity with PFTPs and PFAP offers an advantage in the subsequent recruitment process, and further studies are required to explore this. Free, easily accessible national resources need to be developed to allow early student access and eliminate uneven access to information.

## Appendix

Multimedia Appendix 1 Questions used in the questionnaire.

Multimedia Appendix 2 Illustration demonstrating the relationship between socioeconomic factors and Post-Foundation Training Pathways, Post-Foundation Application Process, and training pathway choices.

## Abbreviations

ACCS: Acute care common stem
BAME: Black, Asian, or Mixed Ethnic
CST: Core surgical training
IMT: Internal medical training
MSRA: Multi-Specialty Recruitment Assessment
PFAP: Post-Foundation Application Process
PFTP: Post-Foundation Training Pathway

## Data Availability

The data sets used and analyzed during this study are available from the corresponding author upon a reasonable request.
