# Supplementary material for: The Impact of UK Medical Students’ Demographics and Socioeconomic Factors on Their Self-Reported Familiarity With the Postgraduate Training Pathways and Application Process: Cross-Sectional Study
Source: JMIR Med Educ. 2023 Nov 24;9:e49013. doi: 10.2196/49013 (PMC10712544; doi:10.2196/49013)
Supplement: Multimedia Appendix 1 [file mededu_v9i1e49013_app1.docx]

**Appendix 1**

**Questions used in the questionnaire**

1. By clicking ‘I agree’ you are consenting to participate in this study and the questionnaire will begin on the next page (Agree/Disagree)

**About you**

2. Which UK Medical School do you attend?

3. What year of Medical School training are you currently in? (Year 1, 2, 3, 4, 5, 6, Intercalation)

4. What is your age?

5. What is your gender? (Male, Female, Non-binary non-conforming, prefer not to say)

6. What is your ethnic group? (BAME (Arab, Asian, Black, Chinese, Hispanic, Indian, Pakistani, Bangladeshi, Mixed, Other BAME), Others, Prefer not to say)

**7.** Do you come from a medical background (defined as having a family member or close friend with a medical degree)?

A .If you come from a medical background, do you think this has facilitated your decision on a career path? (Yes/No)

B. Do you think this has helped you understand post-graduate training pathways?

C. Please expand on the answer above by explaining how coming from a medical background might or might not have facilitated your decision on a career path.

**Medical career pathway**

8. How familiar are you with the training pathway of doctors after graduation?

Familiarity with Post-Foundation Training Pathways (PFTPs) defined as understanding the number of years involved in the desired training pathway and whether the training pathway was run through or required multiple applications (for example, 2 years of core surgical training followed by another application cycle for 4 to 5 years of higher surgical training).

1 – not very familiar

5 – very familiar

9. How familiar are you with the Post-Foundation Training Pathways (PFTPs)?

Familiarity with Post-Foundation Application Process (PFAP) defined as an understanding of the current criteria for candidate selection (for example, use of MSRA, portfolio, interviews) and content of the said criteria (for example, portfolio and interviews assessing qualities such as leadership, academics, teaching).

1 - Not very familiar

5 – Very familiar

10. What training would you like to undertake after Foundation Years? (Internal Medical Training, Core Surgical Training, Neurosurgery, GP Training, Acute Care Common Stem, Psychiatry, Obstetrics and Gynaecology, Radiology, Ophthalmology)

**Suggestions to Improve your understanding of training pathways**

11. What resource would you prefer to improve your familiarity with PFTPs and PFAP?
