# Supplementary material for: The Impact of UK Medical Students’ Demographics and Socioeconomic Factors on Their Self-Reported Familiarity With the Postgraduate Training Pathways and Application Process: Cross-Sectional Study
Source: JMIR Med Educ. 2023 Nov 24;9:e49013. doi: 10.2196/49013 (PMC10712544; doi:10.2196/49013)
Supplement: Multimedia Appendix 2 [file mededu_v9i1e49013_app2.docx]

Illustrations of (A) the relationship between gender and self-reported familiarity with Post-Foundation Training Pathway, (B) self-reported familiarity with Post-Foundation Application Process, and (C) training pathway choices. The number above each bar represents the number of responses received for that category.

Illustrations of (A) the relationship between ethnicity and self-reported familiarity with Post-Foundation Training Pathways, (B) self-reported familiarity with Post-Foundation Application Process, and (C) training pathway choices. The number above each bar represents the number of responses received for that category.

Illustrations of (A) the relationship between medical background and self-reported familiarity with Post-Foundation Training Pathways, (B) self-reported familiarity with Post-Foundation Application Process, and (C) training pathway choices. The number above each bar represents the number of responses received for that category.

Illustrations of (A) the relationship between age and self-reported familiarity with Post-Foundation Training Pathways, (B) self-reported familiarity with Post-Foundation Application Process, and (C) training pathway choices. The number above each bar represents the number of responses received for that category.
